# How do internal medicine subspecialty societies support clinician-educator careers? A qualitative exploratory study

**DOI:** 10.1186/s12909-022-03287-w

**Published:** 2022-03-31

**Authors:** Lekshmi Santhosh, Emily Abdoler, Bridget C. O’Brien, Brian Schwartz

**Affiliations:** 1 Department of Medicine, University of California-San Francisco, CA San Francisco, USA; 2 Department of Medicine, University of Michigan, Ann Arbor, MI USA

**Keywords:** Societies, Subspecialty, Professional development, Clinician-educator, Career development

## Abstract

**Background:**

Internal Medicine (IM) subspecialty professional societies can provide valuable community, recognition, resources, and leadership opportunities that promote career success. Historically, this support focused on clinical and research dimensions of academic careers, but educational dimensions have gained more attention recently. This study explores how IM subspecialty professional societies support their clinician-educator members.

**Methods:**

Using a qualitative study with two phases, the authors collected information from each IM subspecialty society’s website about support for medical education. Using information from the first phase, we developed an interview guide for subspecialty society leaders. We used inductive thematic analysis to analyze interview transcripts.

**Results:**

Website analysis identified various mechanisms used by several IM subspecialty societies to promote medical education. These included websites focused on medical education, dedicated medical education poster/abstract sessions at annual meetings, and strategies to promote networking among clinician-educators. Interviews with eight subspecialty society leaders about the professional societies’ roles with respect to medical education yielded four main themes: [1] varying conceptions of “medical education” in relation to the society [2] strategies to advance medical education at the society level [3] barriers to recognizing medical education [4] benefits of clinician-educators to the societies. Integrating these themes, we describe recommended strategies for professional societies to better serve clinician-educators.

**Conclusions:**

We explore how IM subspecialty societies attend to a growing constituency of clinician-educators, with increasing recognition and support of the career path but persistent barriers to its formalization. These conversations shed light on opportunities for professional subspecialty societies to better serve the needs of their clinician-educator members while also enabling these members to make positive contributions in return.

## Introduction

Although some specialties, such as general internal medicine (GIM) and emergency medicine [1–4], have well-established communities and pathways for clinician-educators, others have only recently recognized the needs of their clinician-educator members. Often, the predominant career pathways for academic internal medicine (IM) subspecialties are based on basic science or clinical/translational research models. Faculty with these traditional career trajectories have benefited from opportunities for mentorship, networking, and clear expectations for advancement and promotion [5]. Efforts to design similar pathways for IM subspecialty clinician-educators have required institutions to grapple with adapting traditional research-based pathways for academic promotions to a model that recognizes the value of clinician-educators [4, 6]. Clinician-educators may feel like “ugly ducklings,” [7] marginalized within a culture of academic medicine that values research over teaching [8]. Given that this is a relatively new career pathway for subspecialists, developing robust communities to support clinician-educator careers in the IM subspecialties is critical [9, 10].

IM subspecialty societies may help fulfill this void by building community and providing opportunities for networking and leadership that foster career success. For example, the Society of General Internal Medicine has advocated for its clinician-educator members for decades, from creating guidelines for promotion and tenure for clinician-educators, to creating a dedicated journal for generalist medical education scholarship, to serving as a research hub to conduct large survey-based scholarship [11]. Similarly, several academic emergency medicine societies have established mentorship structures for clinician-educator members [12]. Efforts by IM subspecialty societies to create similar structures and programs likely would benefit their clinician-educators members, since their career pathway is less well-recognized and they may lack local mentorship and support [13, 14].

Professional subspecialty societies legitimize career paths by serving as “gatekeepers” to critical career and leadership development opportunities for physicians [15, 16]. These societies play crucial roles in defining communities of subspecialty physicians by holding annual conferences to inform members of emerging scholarship and clinical practice changes [17]. These conferences focus on very subspecialty-specific medical knowledge and build specific communities. Additionally, they often promote asynchronous forms of community-building such as list-servs, online communities, and publications. Professional societies thus form natural communities to support their members and “serve to unite members through research and education” [18]. Their leadership and programming play key roles in dictating issues important to the subspecialty.

Traditionally, subspecialty societies have not featured medical education as a distinct discipline, and the exact numbers of clinician-educators in each subspecialty society is unknown. However, some have recently begun to recognize it. For example, the Infectious Disease Society of America (IDSA) progressed from first having an abstract-submission category in medical education for its annual conference in 2015 to creating a dedicated IDSA Medical Education Workgroup in 2016 [10]. Similarly, the American Thoracic Society now boasts a Section on Medical Education comprising over 1600 members and recently founded ATS Scholar, a dedicated journal for medical education research.

Given the variation among societies, we aimed to explore how IM professional subspecialty societies support clinician-educator members. In doing so, we sought to identify strategies for societies to more concretely support subspecialty clinician-educators for successful careers.

## Materials and methods

### Study design

We used a general inductive approach to explore IM subspecialty society support for clinician-educators. We used a two-phase qualitative research study to first collect contextual information about support for education in each subspecialty society. The second phase involved interviews with subspecialty society leaders about the role of medical education in their societies.

### Data collection – phase 1

In the first phase, we examined the websites of subspecialty societies for any information or activities regarding medical education. We focused on the eight largest IM subspecialty societies based on National Residency Match Program application data, since they represent the largest proportion of IM subspecialists: [ 19] cardiology, endocrinology, gastroenterology, hematology, infectious diseases, nephrology, oncology, and pulmonary/critical care. For the purpose of this manuscript, societies/specialties are omitted to maintain anonymity. We used a structured template to systematically document whether societies had medical education websites, journals, conferences, and networking groups. We examined annual meeting agendas from 2018 to 2019 to see if there was programming (e.g. seminars, poster sessions, oral presentations, etc.) related to medical education. We updated this information to document updates in medical education offerings by evaluating both websites and meeting agendas in February 2022 so the most current information is available.

### Data collection – phase 2

The findings from phase 1 provided context for the second phase of our study, during which we conducted semi-structured interviews with subspecialty society presidents. Based on findings from the first phase and informed by concepts embedded in the career-focused mentoring framework for GIM clinician educators [20], we developed nine open-ended questions as prompts, shown in Table 1. Questions focused on the society’s mission, the society’s forms of support for medical education, training for clinician-educators, medical education-specific programming, and the impact of medical education on the society.

**Table 1:** Structured interview questions asked of each society’s leader

1. What is your society’s mission?	
2. What role does medical education play in the mission of your society?	
3. Among the many career pathways of your constituents, is a career in medical education one that your society has recognized?	
4. What does it mean to you to support medical educators?	
5. What does your society do to help your members learn how to be better educators and /or teachers?	
6. What specific programming around medical education does your society have? For example, conferences, lectures at national meeting, journals, contests, etc.	
7. Has supporting medical educators built a sub-community within your society? If so, please describe how.	
8. Has supporting medical educators had an impact, positive or negative, on your society? If so, please describe the impact.	
9. How do you feel your society’s programming for medical education compares to other specialty societies? For example, conferences, lectures at national meeting, journals, contests, etc.	
10. Are there other people you recommend I talk to about medical education in your society?	

### Procedure

One author (LS) contacted each subspecialty society’s president to request interviews, obtained informed consent, and conducted and audio-recorded 40–60 min semi-structured telephone interviews with all participants in 2018. This study was deemed exempt by the University of California-San Francisco Institutional Review Board.

### Analytic approach

Interviews were transcribed and checked for accuracy. We analyzed interview transcripts through an iterative, inductive process consistent with six step process of thematic analysis [21, 22]. One investigator (LS) reviewed transcripts, generated initial codes, and shared the codebook with co-investigator EA. We met to refine codes and coding structure until achieving a final codebook and then independently applied the codes to all transcripts, searched for and documented potential themes in memos, reconciled coding discrepancies through discussion and re-adjudication, and grouped similar coded text together to review, define, and name themes.

### Reflexivity

L.S., E.A., and B.S. are subspecialty clinician-educators. L.S. and E.A. are trained in qualitative research methods and B.O’B. brings expertise in qualitative research and professional development for educators. The authors’ backgrounds as subspecialist clinician-educators influenced their interpretation of the data and, as an outsider, B,O’B. offered a critical lens to these interpretations to enhance clarity and trustworthiness.

## Results

Our review of the websites of the eight largest IM subspecialties is shown in Table 2. Only one subspecialty (Pulmonary) had a dedicated medical education journal. Several societies had dedicated websites to medical education, specific poster and abstract sessions, and well-established mechanisms to promote networking among clinician-educators, such as forming “Sections” or “Communities of Practice” to create smaller sub-communities within the larger society. Most societies employed at least two of these mechanisms to promote medical education.

**Table 2:** Professional subspecialty societies and programming for medical education 2018–19 and updates in 2022

Specialty	Association	Dedicated Med Ed Website	Med Ed Journal	Med Ed Conference	Med Ed Networking	Updates in 2022
*Cardiology*	ACC	Yes	No	No	No	New “Fellows in Training” section on website w/ member networking but not dedicated to medical education
*Endocrinology*	AACE	No	No	No	No	New Fellows Training Series - comprehensive program to support PDs and train fellows, but more focused on ITE
*Infectious Disease*	IDSA	No -- > Yes in 2022	No	No - But dedicated poster & abstract session	IDSA Medical Education Community of Practice (MedEdCOP)	IDSA MedEdCOP includes workgroups d with multiple workgroups
*Gastroenterology*	AGA	Yes	No	No - but in 2022 annual MedEd plenary at national conference	AGA Academy Community Group	AGA Academy of Educators website with more robust offerings like grants
*Nephrology*	ASN	Yes	No	No	Yes - listserv	ASN Website with more robust modular online curricula for remote learning
*Oncology*	ASCO	No	No	No	New in 2022 - Education Scholars program focused on medical education	In 2019 ASCO started new Education Scholars program for MedEd
*Pulmonary*	ATS	Yes	Yes	No - But dedicated seminars & posters	ATS Section on Medical Education	ATS Section on Medical Education website now with more grants, active social media presence, more awards
*Rheumatology*	ACR	No -- > Yes in 2022	No	No -- > Yes in 2022, ACR Education Exchange	No	New ACR Education Exchange Conference focusing on Fellows-in-Training, Educators, PDs, New Rheum2Learn website with educational modules

From our interviews with society leaders about the professional society’s role in medical education, we identified four main themes: (1) varying conceptions of “medical education” (2) strategies to advance medical education (3) barriers to recognizing medical education (4) benefits of clinician-educators to the societies.

### Varying conceptions of “medical education” in relation to the subspecialty society

Subspecialty society leaders conceptualized “medical education” in a variety of ways. The majority viewed medical education as a method to help faculty create educational content for member learning, including content for annual educational conferences, journals, and continuing medical education.

Some leaders viewed medical education as a source of patient education materials or patient care-related documents, such as clinical practice guidelines, while others viewed the role of medical education as defining competencies for larger regulatory bodies.

*“Our education committee [works] in terms of defining curricula and defining regulatory standards in terms of our fellows.”* [Society Leader 6]

Only two societies viewed medical education as a distinct discipline, with clinician-educators focusing on medical education as a career trajectory.

“*We’ve asked our members to self-identify constituency – clinical practice, clinical science, and basic science. We recognized that we have a lot of medical educators that don’t quite fit [ …*]’ *We’re actually expanding the definitions of our constituency to formally recognize people who have chosen medical education as a career.”* [Society Leader 8]

### Strategies to advance medical education at the subspecialty society level

Subspecialty society leaders identified multiple strategies that societies used to advance medical education, including forums for community-building, promoting educational scholarship, formal recognition for educators, faculty development programming, and educating society members via curricula such as continuing medical education (CME).

Some leaders identified specific networking events dedicated to educators:

*“There’s a community who go to the … educators forum [...]. ‘Do you have a good handoff tool, do you want to share it with me, what’s your email?’ The forum becomes a great place for networking. At this time, having it be a more informal community of practice for networking is the way to go. We do have a PD [program director] association. But this is for everyone.”* [Society Leader 8]

Some societies conferred educators with formal recognition, including named awards for educators, dedicated pathways, educator representation on key committees, and grants for educational research.

One society had an annual award providing funding for education research and advanced educational training; some presidents noted that grants and awards helped legitimize the role of clinician-educators in the society.

*“[These awards] promote the professionalization of that track … it’s raised the image or professionalism and visibility within the specialty … People who have received this award – [it’s a] launching pad for the career – they become clerkship directors, associate deans, division chairs, it certainly helps raise visibility that way.”* [Society Leader 2]

Many societies sponsored faculty development for educators, including embedding this content in programming directed towards program directors (PDs).

*“Definitely at the training PD retreat, there are multiple sessions on specific aspects of training, including fellow evaluation, development of unique tools for helping fellows learn.”* [Society Leader 3]

Other societies housed this content within the annual society conference, open to all attendees.

*“We have special education sessions built into our education meeting. How do you do [flipped] classroom? How do you give feedback? How do you be the best teacher you can? We have a committee that works on curricula and webinars … How do you deal with a fellow in clinic? There are also ones on writing letters of recommendation and mentorship.”* [Society Leader 8]

Some societies harnessed clinician-educators to provide curricula for the society, thus curating medical education content experts, who in turn help the society’s membership at large.

“*… Another way of involving people interested in pursuing education and engaging them and their expertise in helping to develop a fellowship curriculum. We get them involved in test-writing or question-writing … We have [CME] … so we involve a lot of people in writing these questions.”* [Society Leader 7]

### Barriers to recognizing medical education

Not all society leaders saw the need to recognize medical education as a specific track; some viewed education as distinct from research or clinical care, or saw societies as not the best venues for this work.

For example, one society leader noted:

*“I don’t think we have any kind of formal recognition [for careers in medical education]. We value leaders in education. But we also value … clinicians. Recognize is a little bit of a charged term … I think the society may help understand and promote aspects just as if [education] were research … I can’t say medical education seems to warrant, or I have not heard of, a need outside the Ed [ucation] committee or PD committee.”* [Society Leader 6]

Another noted how education was separate from research:

*“At our national meeting,[education] is not a big part of the meeting because it’s a scientific meeting that draws 5000 abstracts and stuff like that.”* [Society Leader 7]

Some society leaders recognized barriers that clinician-educators uniquely face and, in some cases, mentioned how societies were trying to remedy the gap:

*“Education has been chronically underfunded in academic medicine. This award is one mechanism that provides funding for people [by providing protected salary support and professional development funds for a clinician-educator].”* [Society Leader 2]

### Benefits of clinician-educators to subspecialty societies

Society leaders recognized how clinician-educators benefited societies, including by building a community of clinician-educators, recruiting future trainees, promoting enthusiasm for the specialty, creating educational content for the society, and educating society members.

Society leaders appreciated formal and informal networking opportunities for educators who were *“largely volunteers who come together to learn how to teach and to support each other.*” [Society Leader 4] Educators were thus able to connect and collaborate on scholarly projects or curriculum development, thus reaping further benefits for the society.

They also recruited future workforce to the field, through programs focusing on pre-college and pre-medical students who would be *“exposed to excellent role models.*” [Society Leader 3] Educators effectively promoted energy and enthusiasm in the specialty, through activities such as *“knowledge bowls, where teams compete.”* [Society Leader 2].

Society leaders appreciated *“new educational tools”* such as self-assessment tools and self-directed learning curricula developed by faculty that were *“nothing short of outstanding. It generates a lot of excitement.”* [Society Leader 3].

Many society leaders engaged clinician-educators for activities such as Board review [Society 3] and self-assessment courses [Society 7]. Other societies used workshops to teach faculty and program directors how to teach, thus aiming for a “*trickle-down approach by training PDs who will hopefully improve med ed in their programs.*” [Society Leader 7].

## Discussion

In this study, we examined the medical education-related content, programming, and infrastructure provided by subspecialty societies, and interviewed society leaders to explore how societies support their clinician-educator members. Based on these findings, we recommend strategies for improving the integration of medical education and support of clinician-educators within IM subspecialty societies. Both an understanding of the ways societies are currently supporting clinician educators and recognition of the barriers clinician educators face should guide development of appropriate clinician educator-focused initiatives within IM subspecialty societies.

Society leaders conceptualized “medical education” in different ways, with views ranging from medical education as a distinct discipline and career path to viewing it as a regulatory requirement. Despite barriers to society leaders recognizing medical education, they acknowledged ways that clinician-educators benefited their societies, thereby legitimizing their roles. Rarely, some society leaders felt.

### Aligning leadership perceptions with society efforts

Our review of societies’ websites revealed that occasionally leadership’s perception of medical education did not necessarily align with the society’s medical education programming. For example, some society websites reported well-developed medical education communities and international conference offerings, yet presidents were not aware of these activities. This disconnect between leadership and society activities reflects leaders being unaware of the full extent of a society’s efforts which can be due to many unknown reasons. However, it could also indicate a society’s larger lack of recognition of clinician-educators, further hindering their career development. Often, society leaders seemed to lack full understanding of the concept of a clinician-educator career, in stark contrast to the leaders’ easy recognition of more traditional basic science or clinical-research pathways.

Literature both within and outside of healthcare [23, 24] has linked leadership support of activities to wellbeing and productivity; this is particularly important as leaders recognize diverse career pathways of their constituents, especially newer and developing pathways. However, leadership support and resources may not necessarily correlate to the experiences of clinician educators, especially if they are actively engaging in a sub-community of educators regardless of leadership awareness of these activities. If leaders better understood what clinician-educators do, then they could help understand the support structures needed to advance in their careers.

### Conceptualizing “clinician-educators”: faculty development as a virtuous cycle

Despite barriers faced by clinician-educators, societies can offer valuable support to help clinician-educators thrive by supporting career development. Since clinician-educators may face challenges attaining recognition at their local institutions, professional subspecialty societies can legitimize clinician-educator career pathways and assist with local recognition. Some societies were quite successful in developing and taking advantage of a community of educators by providing a safe space for educators, encouraging development of educational content, and promoting faculty development – both of clinician-educators and by clinician-educators. For example, we found that the leaders of Society 2 and Society 8 understood the unique professional identity of clinician-educators, and those societies also had more dedicated medical education programming at annual conferences.

Some society leaders realized that supporting a community of clinician-educators could be a mutually beneficial symbiotic relationship. Educators would not only develop educational content to educate the society’s larger community, but also create a community of educators leading to a “virtuous cycle” of further educational and scholarly opportunities. Additionally, promoting networks within societies can enable clinician-educators to build relationships external to their institutions, which can assist in obtaining letters of support for the advancement and promotions process, as additive benefits to the faculty members.

### Strategies for subspecialty societies to support clinician-educator careers

We found that subspecialty societies with programming for clinician-educators mirrored many of the strategies outlined by structured career mentoring programs in GIM. Specifically, GIM career mentoring programs noted that successful mentorship led to improved career management self-efficacy, mentoring and job satisfaction and scholarship [4]. Subspecialty societies that had more programming for clinician-educators also employed strategies focused on mentoring, including formal networking structures, improved self-efficacy and well-being by encouraging community-building, and promoting educational scholarship through grants, posters, and oral presentations.

Based on our structured interviews, Table 3 showcases four strategies that societies can employ to support clinician-educators: community-building events, faculty development and curricular content for member learning, support for educational research, and recognition and prestige for educators. Some societies had multiple of these components, while others had few of these components.

**Table 3:** Recommended strategies for supporting medical education at the professional society level

Community Building	Education Research	Recognition and Prestige	Faculty Development
Online listservs Discussion forums Networking events Mentorship programs	Dedicated Symposia Dedicated Abstract Category Dedicated Journal/Issue Educational Grants	Mission Statement Named Awards Dedicated Pathway/Track Integration with Larger Society	Teaching Workshops Certificate Programs Curriculum Development by Educators

### Limitations

We interviewed subspecialty society leaders during one academic year (2019), which may not reflect current subspecialty society offerings. Presidential terms can be brief and society’s culture and support for educators may shift as leadership changes. We interviewed society presidents who, admittedly may not be aware of all medical education offerings. Nonetheless, we believe their perspectives provide a barometer of how societies perceive their clinician-educators. This work focuses on international subspecialty societies, though the majority of members are U.S. participants. While this may be a limitation of our work, to our knowledge, there is no literature on the role of IM subspecialty societies in other nations. Further research should explore perspectives of clinician-educators embedded in various U.S. and international societies and discuss how they experience formal and informal support structures both within and outside their professional communities.

## Conclusions

Our findings explore how IM subspecialty societies attend to a growing constituency of clinician-educators. These conversations shed light on opportunities for subspecialty societies to better serve clinician-educators, while enabling them to contribute to their societies. Clinician-educators can symbiotically contribute to a virtuous cycle that can benefit societies and educators’ own career development.

## Data Availability

There were no proprietary data/materials used for this study. Data sharing: The corresponding author Dr. Santhosh can be contacted if further raw data are required.
